# Editorial: Factors Underpinning and Influencing Drivers' Aberrant Behaviors Across the Life Course

**DOI:** 10.3389/fpsyg.2019.03030

**Published:** 2020-01-22

**Authors:** Fabio Lucidi, Andrea Bosco, Luca Mallia, Annalisa Setti

**Affiliations:** ^1^Department of Social and Developmental Psychology, Sapienza University of Rome, Rome, Italy; ^2^Department of Education Science, Psychology, Communication Science, University of Bari Aldo Moro, Bari, Italy; ^3^Department of Movement, Human and Health Sciences, University of Rome “Foro Italico”, Rome, Italy; ^4^School of Applied Psychology, University College Cork, Cork, Ireland

**Keywords:** driving, age, personality, violations, errors, lapses, cognitive processes, protective factors

## Introduction

Human factors play a fundamental role in driving performance and in the errors and violations that can be committed behind the wheel. While new technologies will soon allow “*automatic*” driving, it continues to be crucial to analyse and understand the factors determining human error during driving. Errors, carelessness, and driving violations can be linked to age and experience, as well as to the driver's internal characteristics (e.g., personality, cognitive, affective, and psychophysiological processes). Such characteristics can be either stable, or variable, i.e., dependent on the state of the individual, and in turn, they interact with contextual aspects–that is, the social environment, such as the presence of passengers, or the physical environment, including the type of road, and the condition of the vehicle. Such characteristics and specific processes, as highlighted in this Research Topic, are peculiar to specific age groups, while others are more general and studied over the course of the lifespan. Given the complexity of the topic, it is not surprising that it is addressed in the scientific literature using diverse methodological approaches and measurement tools to account for these different dimensions.

This Research Topic is intended to provide an overview of empirical studies referring to both internal and external factors related to driving performance and driving errors, and it is articulated in different age groups. The studies collected here address different issues and use different methodologies, providing an overview of the complexity and richness of this field.

## Studies Involving Young Drivers

A first set of studies in the present special topic concerns young drivers, two studies look at young moped drivers, and five studies investigate young car drivers.

For young moped drivers, the focus in the literature has been on analyzing specific risk factors (e.g., personality) that can negatively impact driving performance by increasing the risk of errors, lapses, and violations. Lucidi et al. identified three personality sub-types (i.e., risky, worried, and careful moped riders) on a large sample of adolescent moped drivers, who also differ significantly in risky driving behaviors, attitudes toward traffic safety, risk perception, and self-reported accident involvement. The results empirically support the notion that certain combinations of personality characteristics are associated with risky driving in moped riders. The study by Gianfranchi et al., on the other hand, identified three different profiles (Imprudent, Prudent, and Insecure) on the basis of driving performance of inexperienced young people in a virtual driving environment. The results of the study showed that these three groups also present different combinations of personality traits and beliefs, empirically confirming the close and bidirectional relationship between the behavioral and personality profiles of young moped drivers.

Lazuras et al. studied young car drivers, and examined the impact on risky driving of two “clusters” of individual characteristics, on the one hand those defined as “hot,” such as the impulsivity and sensation seeking, and on the other those defined as “cold” such as self-regulation and emotional regulation. An important finding was the mediation of self-regulation (“cold”) in the relationship between sensation seeking (“hot”) and self-reported errors, indicating that “hot” and “cold” individual characteristics are somewhat integrated in predicting self-reported driving behaviors. The study by Simon-Morton et al. focuses on an important issue in the literature, namely the effects of passenger presence on driving performance in young drivers. More specifically, the two studies carried out in a simulation context revealed that both male and female teenagers are influenced in their risk driving behavior by the attitude of passengers regarding risk-acceptance and associated potential distress experienced as an effect of social exclusion.

Three studies investigated basic cognitive processes and neurophysiological correlates of young drivers' performance. The study by Cassarino et al. looked at attentional demands depending on the road, urban or rural; they found that in a short simulated drive, the urban road was not more demanding than the rural road for these younger drivers. The study by Yan et al. utilized machine learning to distinguish between aggressive and conservative drivers based on power spectral density of the Elecroencephalography (EEG), showing distinct brain activity in these two types of drivers at different frequency bands. Finally, the study by Ding et al. sought to identify and analyse the neuro-physiological correlates of driving in a driving simulator and to relate them with the personality of drivers, thus integrating measures obtained with different methodologies (i.e., EEG and questionnaires).

## Studies Involving Adult and Older Drivers

A second set of studies focused on adult drivers with the purpose of evaluating the basic cognitive processes related to driving and the corresponding neurophysiological correlates. The review by Palmiero et al. summarizes empirical data related to the effects that a secondary task typically has on driving performance, as well as the corresponding neurophysiological correlates. The review indicated that there is substantial consensus across studies on occipital lobe deactivation and fronto-temporal lobe activation associated to the attentional shifting from driving to a secondary task, even in absence of evident modification of driving performance. However, neuroimaging studies present a series of methodological flaws; the authors also indicated personality as useful dimension to explore in relation to the attentional profile of individuals. The study by Yan et al. looked at a sample of adult drivers to analyse the complex relationships between drivers' behavior, studied with the driving simulator, drivers' personality characteristics, evaluated through self-reported questionnaires, and the neurophysiological activation, evaluated through EEG. The authors suggested that information on driving style gathered through those different methods should be integrated into advanced driving assistance system (i.e., ADAS) in order to anticipate risky driving behavior.

Finally, a third set of studies focused on the analysis of psychological processes and individual factors typically associated with driving performance in older drivers. The study by Spano et al. provided a contribution to the factorial validation of three high-reputation questionnaires on driving behavior, namely, the Driver Behavior Questionnaire, the Attitudes Toward Traffic Safety, and the Driving Mobility Questionnaire. A complex statistical analysis based on hurdle model showed that all sub-factors of Driver Behavior Questionnaire predicted the likelihood of self-reported road collisions (both as unique or multiple events) in a sample of older participants. Gormley and O'Neill, on the other hand, analyzed the role that driving, in terms “mobility,” has for the older adults, highlighting its effects on their quality of life. Over 8,000 individuals were surveyed in the Irish Longitudinal Study on Ageing (TILDA) and the results indicated that men keep driving for longer than women; driving was more frequent in married participants and had a positive impact on quality of life and loneliness. The study by Saryazdi et al. directly compared older drivers with young drivers on their attention to the visual scene while driving, specifically “Inattentional Blindness,” i.e., lack of awareness of people or objects at the side of the road. Both younger and older participants experienced inattentional blindness. However, while younger adults improved their performance across the experiment, the same did not occur for older, potentially indicating slower learning from experience.

## Conclusions

This Research Topic highlights the importance to consider contextual and subjective human factors in driving, which impact on performance and co-determine safe driving, together with well-known factors such as experience. These factors, studied with different methods, should not be overlooked in understanding driving behavior.

## Author Contributions

All the authors have substantially contributed to the development and preparation of the editorial. Furthermore, all authors have approved the final version of the manuscript. The authors have agreed to be accountable for all aspects of the manuscript in ensuring that questions related to the accuracy or integrity of any part of it are appropriately investigated and resolved.

### Conflict of Interest

The authors declare that the research was conducted in the absence of any commercial or financial relationships that could be construed as a potential conflict of interest.

